# Impact of sedative and appetite-increasing properties on the apparent antidepressant efficacy of mirtazapine, selective serotonin reuptake inhibitors and amitriptyline: an item-based, patient-level meta-analysis

**DOI:** 10.1016/j.eclinm.2024.102904

**Published:** 2024-11-07

**Authors:** Fredrik Hieronymus, Alexander Lisinski, Elias Eriksson

**Affiliations:** a Department of Pharmacology, Institute of Neuroscience and Physiology, Sahlgrenska Academy, University of Gothenburg, Gothenburg, Sweden; b Department of Psychiatry, Sahlgrenska University Hospital, Region Västra Götaland, Gothenburg, Sweden

**Keywords:** Mirtazapine, SSRIs, SNRIs, Amitriptyline, Meta-analysis, Depression

## Abstract

**Background:**

In an influential network meta-analysis, the tricyclic antidepressant (TCA) amitriptyline was found to be the most efficacious of 21 antidepressants, hence outranking selective serotonin reuptake inhibitors (SSRIs) and serotonin and noradrenaline reuptake inhibitors (SNRIs). The alpha_2_/5HT_2A_/_2C_/_3_/H_1_ antagonist mirtazapine was ranked as the second most effective and appeared at least as effective as the SSRIs and SNRIs that followed next. Since the most common effect parameter in depression trials—the sum score of the Hamilton Depression Rating Scale (HDRS-17-sum)—includes three items measuring sleep and two measuring appetite and weight, this outcome could be the result of amitriptyline and mirtazapine being more sedative and orexigenic. The main aim of this study was to compare mirtazapine with SSRIs or amitriptyline with respect to impact on core depression symptoms.

**Methods:**

Access to patient-level data from all company-sponsored, acute-phase, HDRS-based, and randomized trials of mirtazapine in adult major depression available to Merck was granted. Thirty-two studies compared mirtazapine to placebo and/or amitriptyline or an SSRI whereas five compared mirtazapine to another TCA or an SNRI, venlafaxine. Data were divided into subgroups for direct comparisons of mirtazapine vs placebo or different subgroups of antidepressants. Indirect comparisons of SSRIs vs amitriptyline were also undertaken. Mixed models for repeated measures were used to assess efficacy as reflected by i) HDRS-17-sum, ii) six core depression symptoms (HDRS-6-sum), and iii) all individual items.

**Findings:**

The dataset consisted of 5974 participants. Mirtazapine (n = 1362) outperformed SSRIs (n = 1369) on HDRS-17-sum, but this was due to differences regarding items reflecting sleep, appetite, and gastrointestinal dysfunction—with respect to reducing depressed mood, suicidality, and psychic anxiety, SSRIs and/or venlafaxine were more effective. Amitriptyline (n = 622) was superior to mirtazapine (n = 606) in reducing depressed mood, and the combined group of all TCAs (n = 831) outperformed mirtazapine (n = 824) also with respect to other core depression symptoms. Since there were no head-to-head comparisons of amitriptyline vs SSRIs, no firm conclusion may be drawn with respect to relative efficacy of the two, but indirect comparisons support the notion that amitriptyline and other tricyclics may be superior also to SSRIs.

**Interpretation:**

While the apparent superiority of mirtazapine over SSRIs is explained by its sedative and orexigenic properties, and by its absence of gastrointestinal side effects, amitriptyline appeared more effective in reducing core symptoms of depression than mirtazapine and possibly also than SSRIs; given the indirect nature of the latter comparison, this outcome should however be interpreted with caution. Lack of information regarding dosing was another important limitation. The study illustrates the value of item-based analyses when assessing the relative efficacy of antidepressants.

**Funding:**

The Swedish Research Council, the Swedish Brain Foundation. The Gothenburg Society of Medicine, the Swedish Society of Medicine, Åke Wiberg's Foundation, Märta Lundqvist's Foundation, Fredrik and Ingrid Thuring's Foundation, Söderström-Königska Foundation and Frimurare-Barnhusdirektionen in Gothenburg.

**Research in context**

**Evidence before this study**

In 2018, Cipriani and colleagues published a comprehensive and influential network meta-analysis in which amitriptyline and mirtazapine were found to be the two most efficacious antidepressant out of the 21 included. Mirtazapine was second on the list but did not appear significantly better than the selective serotonin reuptake inhibitors (SSRIs) and serotonin and noradrenaline reuptake inhibitors (SNRIs). This study was based on the primary efficacy parameters applied in the included trials, which has usually been the sum score of the 17 items of the Hamilton Rating Scale (HDRS-17-sum). However, since the HDRS includes three items reflecting sleep and two items reflecting appetite, the prominent position of amitriptyline and mirtazapine might be explained by both substances displaying marked sedative and orexigenic properties. Since the development program for mirtazapine includes studies where mirtazapine was compared to SSRIs and to amitriptyline, this data set can be used to address this possibility, hence providing a fuller understanding of possible differences between these three drugs with respect to antidepressant efficacy.

**Added value of this study**

Based on a patient-level, item-based meta-analysis including 32 trials where mirtazapine had been compared with placebo, an SSRI, or amitriptyline, we report that the apparent superiority of mirtazapine over SSRIs with respect to reduction in HDRS-17-sum can be explained by mirtazapine improving sleep, increasing appetite, and causing less gastrointestinal side effects; in contrast, SSRIs were more effective than mirtazapine in reducing depressed mood. Like mirtazapine, amitriptyline produced a marked improvement in sleep, but also outperformed mirtazapine at endpoint with respect to depressed mood. An indirect comparison of amitriptyline and SSRIs—which should be interpreted with caution since the outcome may be confounded by systematic differences in trial design—lent some support for amitriptyline being superior also to SSRIs. Analyses of five trials comparing mirtazapine to either venlafaxine or a tricyclic antidepressant other than amitriptyline yielded results largely in line with the outcomes of amitriptyline and SSRI trials.

**Implications of all the available evidence**

In line with the Cipriani network meta-analysis and several head-to-head comparisons, the present study does not rebut the notion that amitriptyline displays superior efficacy compared to other antidepressants. In contrast, the apparent superiority of mirtazapine over SSRIs when efficacy assessments are based on the HDRS-17-sum appears to be solely a reflection of mirtazapine improving sleep, increasing appetite, and not being marred by gastrointestinal side effects. With respect to reducing depressed mood, as well as other affective and cognitive symptoms of depression, SSRIs and SNRIs instead appear more rather than less effective than mirtazapine. Considering the substantial heterogeneity both in the presentation of depressive disorder and in the pharmacodynamics profiles of different antidepressants, symptom-specific analyses may provide a path toward more refined treatments as well as better matching of patients to treatments than the currently used rating-scale model.

## Introduction

Clarifying if there are differences in efficacy between antidepressants may both guide prescribing clinicians and facilitate the development of refined medications. An important step was thus taken by Cipriani and co-workers when comparing 21 antidepressants in a comprehensive network meta-analysis.^1^ In this study, amitriptyline and mirtazapine were ranked first and second in terms of efficacy, i.e., above the more commonly prescribed selective serotonin reuptake inhibitors (SSRIs) and serotonin and noradrenaline reuptake inhibitors (SNRIs). The difference between mirtazapine and the SSRIs and SNRIs was, however, small and of unlikely significance.

This outcome is of theoretical interest since amitriptyline, mirtazapine, SSRIs, and SNRIs differ in their pharmacodynamics profiles. Amitriptyline is a tricyclic antidepressant inhibiting serotonin and noradrenaline transporters (SERT and NET) and antagonizing serotonergic (e.g., 5-HT_2A_), histaminergic (H_1_), muscarinic, and noradrenergic (alpha_1_) receptors.^2^ Mirtazapine is an antagonist at adrenergic alpha_2_-adrenoceptors, histaminergic H_1_-receptors, and serotonergic 5-HT_2A_, 5-HT_2C_, and 5-HT_3_ receptors, with enhanced noradrenaline release following blockade of alpha_2_-adrenergic autoreceptors often suggested as the likely mechanism of action.^3^ SSRIs and SNRIs, in contrast, are not strong antagonists vis-à- vis any transmitter receptors but only act as inhibitors of SERT, and SERT plus NET, respectively.

The most common efficacy parameter in depression trials has been the reduction in the sum score of the 17-item version of the Hamilton Depression Rating Scale (HDRS-17-sum).^4^ Since three HDRS items reflect sleep and two reflect appetite and weight, a drug improving sleep and/or enhancing appetite may cause an apparent reduction in depression severity independently of any actual antidepressant effect. Both amitriptyline and mirtazapine displaying sedative and orexigenic properties^2^^,^^3^ hence raises the question of to what extent this difference may explain why the former, and possibly the latter, come out as superior compared to the SSRIs and SNRIs with respect to HDRS-17-sum-assessed efficacy.^1^

The development program for mirtazapine mainly included trials comparing it to placebo, SSRIs, or amitriptyline. To a lesser extent, three other TCAs and one SNRI also served as comparators. These studies hence lend themselves well to assessing how differences in sedative and appetite-increasing effects translates into apparent antidepressant efficacy. In this patient-level meta-analysis, we assess how all 17 individual symptoms rated by the HDRS-17 develop during six weeks acute-phase treatment for depression for the studied antidepressants (and placebo).

## Methods

### Data acquisition

We searched for double-blind, HDRS-based, placebo- or active comparator-controlled, company-sponsored phase II-IV trials of mirtazapine for the acute-phase treatment of depression in the FDA Drug Approval Package for mirtazapine^5^ as well as in previous meta-analyses.^1^^,^^6^, ^7^, ^8^, ^9^ This generated a list of 49 trials which were deemed likely to meet our inclusion criteria (Supplementary Table S1). Individual patient data for these 49 trials were requested from Merck Sharp & Dohme (MSD) (Rahway, NY, USA) on Jan 31, 2021, and we also asked for patient-level data from any possible additional trial matching our inclusion criteria that might have escaped our attention. The sources we used to identify trials likely to meet our inclusion criteria varied with respect to how detailed the trials were described and did not use homogeneous trial identifiers. Hence, out of 49 requested trials, two (E−1659 and Wheatley 1998; see Supplementary Table S1) turned out to be duplicates, one (E−1627) was an open-label trial and yet another (36801) could not be identified by MSD. For three other trials, which were academic but supported by grants from MSD, data could not be retrieved as they were likely not in the possession of MSD. We thus received individual patient data from 42 unique trials. Of these, two trials turned out to not have used the HDRS and were hence excluded. Two studies comparing mirtazapine to maprotiline and one comparing it to trazodone were also excluded, as were trial arms of trazodone and combined mirtazapine and paroxetine.

Included active comparators were the tricyclic antidepressants (TCAs) amitriptyline, clomipramine, doxepin, and imipramine, the SSRIs fluoxetine, fluvoxamine, paroxetine, and sertraline, and the SNRI venlafaxine. Four amitriptyline trials were conducted in inpatients, five in outpatients, and one in both in- and outpatients. The clomipramine, dothiepin, and imipramine trials were all in inpatients. Seven SSRI trials were conducted in outpatients and one in both in-and outpatients; for the remaining six SSRI trials, information on setting was unavailable. One venlafaxine trial was undertaken in outpatients and the other in inpatients (Table 1).

**Table 1:** Baseline characteristics for all included studies.

Protocol	Treatment (n)	Age	% Female	HDRS-17	HDRS-6	Depressed mood	Trial length	Week 6 assessment	In-/outpatient
1900001	Mirtazapine 15–45 mg (186) Fluoxetine 20–60 mg (199) Placebo (66)	41.2 (11.9)	59%	21.8 (3.7)	11.8 (1.8)	2.7 (0.6)	8	No	Outpatients
1900002	Mirtazapine 30 mg (21) Paroxetine 20 mg (21) Mirtazapine 15 mg + Paroxetine 10 mg (19)^a^	42.8 (10.6)	48%	23.9 (4.3)	12.0 (2.2)	3.0 (0.5)	8	Yes	N/A
1900003	Mirtazapine 20–60 mg (72) Amitriptyline 75–225 mg (71)	44.5 (10.6)	73%	22.6 (3.9)	10.7 (2.1)	2.7 (0.5)	5	No	Inpatients
1900004	Mirtazapine 15–45 mg (114) Sertraline 50–200 mg (122)	43.5 (11.9)	64%	22.4 (3.1)	12.2 (1.9)	2.7 (0.7)	8	Yes	N/A
1900005	Mirtazapine 30–45 mg (58) Fluoxetine 20–40 mg (57)	47.7 (15.0)	63%	24.0 (5.3)	11.3 (2.8)	2.9 (0.8)	6	Yes	N/A
1900007	Mirtazapine 5–35 mg (49) Placebo (48) Trazodone 40–280 mg (47)^a^	62.0 (6.3)	54%	22.0 (3.3)	11.6 (1.8)	2.6 (0.6)	6	Yes	Outpatients
1900008	Mirtazapine 15–60 mg (38) Placebo (12)	42.9 (11.8)	38%	21.3 (4.5)	12.4 (2.6)	2.6 (0.6)	5	No	N/A
1900010	Mirtazapine 5–35 mg (44) Amitriptyline 40–280 mg (47) Placebo (48)	45.5 (11.9)	56%	21.4 (3.3)	11.0 (1.8)	2.4 (0.6)	6	Yes	Outpatients
1900011	Mirtazapine 15–45 mg (65) Paroxetine 20–40 mg (69)	44.4 (12.6)	69%	25.1 (5.0)	12.1 (2.7)	2.8 (0.8)	6	Yes	N/A
1900012	Mirtazapine 15–45 mg (200) Placebo (65)	41.1 (11.1)	63%	22.1 (3.5)	12.3 (1.9)	2.8 (0.5)	8	Yes	Outpatients
1900013	Mirtazapine 20–60 mg (79) Doxepin 50–300 mg (74)	41.1 (10.8)	59%	22.2 (3.8)	11.6 (2.1)	2.7 (0.5)	6	Yes	Inpatients
1900015	Mirtazapine 20–60 mg (122) Amitriptyline 75–225 mg (124)	47.0 (10.7)	78%	25.9 (4.7)	12.0 (2.6)	2.7 (0.6)	6	Yes	Inpatients
1900018	Mirtazapine 30–45 mg (89) Paroxetine 20–30 mg (81)	39.7 (12.8)	73%	24.1 (3.7)	12.0 (1.8)	2.7 (0.5)	8	Yes	N/A
1900019	Mirtazapine 15–60 mg (61) Fluoxetine 20–40 mg (65)	47.2 (15.0)	56%	26.3 (4.5)	12.7 (2.5)	2.9 (0.6)	6	Yes	N/A
1900020	Mirtazapine 5–35 mg (49) Placebo (50) Amitriptyline 40–280 mg (49)	38.1 (11.6)	68%	27.5 (4.2)	12.8 (1.7)	2.9 (0.6)	6	Yes	Outpatients
1900021	Mirtazapine 20–80 mg (87) Clomipramine 50–200 mg (86)	50.7 (12.4)	72%	25.7 (5.2)	13.4 (2.6)	3.1 (0.5)	6	Yes	Inpatients
1900022	Mirtazapine 15–45 mg (12) Paroxetine 20–40 mg (13)	42.9 (11.9)	52%	23.5 (3.6)	11.6 (1.9)	2.5 (0.5)	6	Yes	N/A
1900023	Mirtazapine 15–45 mg (54) Amitriptyline 30–90 mg (59)	70.8 (5.5)	82%	23.8 (4.3)	11.9 (2.4)	2.7 (0.6)	6	Yes	Outpatients
1900024	Mirtazapine 15–50 mg (41) Placebo (43)	46.8 (14.3)	50%	23.9 (4.3)	12.3 (2.0)	2.8 (0.7)	6	Yes	Inpatients
1900025	Mirtazapine 15–45 mg (123) Paroxetine 20–40 mg (107)	71.8 (5.5)	51%	22.4 (3.5)	11.6 (2.0)	2.9 (0.7)	8	Yes	Outpatients
1900026	Mirtazapine 30–45 mg (95) Fluvoxamine 50–150 mg (98)	41.3 (13.1)	44%	22.6 (3.7)	11.2 (2.0)	2.3 (0.7)	6	Yes	N/A
1900027	Mirtazapine 30–60 mg (15) Amitriptyline 113–150 mg (15) Placebo (15)	37.5 (5.5)	48%	30.6 (4.4)	16.8 (1.9)	3.5 (0.5)	6	Yes	Inpatients
1900028	Mirtazapine 15–45 mg (98) Fluvoxamine 50–150 mg (101)	40.5 (12.3)	67%	25.1 (3.9)	12.9 (2.1)	2.9 (0.5)	6	Yes	N/A
1900029	Mirtazapine 15–60 mg (145) Fluoxetine 20–40 mg (147)	43.7 (11.6)	72%	28.6 (3.0)	14.0 (1.7)	3.3 (0.7)	8	Yes	N/A
1900030	Mirtazapine 5–60 mg (110) Placebo (28)	40.1 (11.8)	66%	23.1 (3.0)	12.7 (1.5)	2.9 (0.3)	6	Yes	Outpatients
1900031	Mirtazapine 20–60 mg (71) Amitriptyline 75–225 mg (73)	47.3 (11.4)	78%	27.1 (4.2)	13.4 (1.9)	3.0 (0.6)	6	Yes	Inpatients
1900032	Mirtazapine 5–35 mg (39) Amitriptyline 40–280 mg (38) Placebo (37)	43.7 (13.3)	50%	24.6 (4.9)	12.7 (2.3)	2.8 (0.6)	6	Yes	Outpatients
1900033	Mirtazapine 15–45 mg (124) Paroxetine 20–40 mg (121)	47.3 (10.5)	64%	22.4 (3.3)	11.3 (2.0)	3.0 (0.8)	6	Yes	Outpatients
1900034	Mirtazapine 30–45 mg (125) Venlafaxine 75–225 mg (115)	45.6 (12.1)	56%	24.7 (2.8)	12.3 (1.9)	3.0 (0.7)	6	Yes	Outpatients
1900035	Mirtazapine 5–35 mg (42) Placebo (45)	42.5 (10.7)	57%	23.7 (3.7)	12.4 (2.0)	2.8 (0.4)	6	Yes	Outpatients
1900036	Mirtazapine 20–60 mg (63) Placebo (61)	41.2 (11.8)	67%	23.1 (3.8)	11.4 (2.0)	2.3 (0.5)	5	No	N/A
1900037	Mirtazapine 5–35 mg (48) Amitriptyline 40–280 mg (48) Placebo (48)	42.2 (11.4)	56%	23.4 (3.6)	12.7 (1.9)	2.7 (0.5)	6	Yes	Outpatients
1900038	Mirtazapine 5–35 mg (44) Placebo (44)	39.6 (9.0)	51%	22.1 (3.1)	11.8 (1.3)	2.9 (0.5)	6	Yes	Outpatients
1900039	Mirtazapine 20-mg (52) Imipramine 75-mg (49)	46.6 (10.7)	79%	26.3 (4.6)	11.6 (2.7)	3.2 (0.7)	6	Yes	Inpatients
1900040	Mirtazapine 30–45 mg (171) Sertraline 50–150 mg (168)	41.6 (11.9)	58%	24.6 (4.4)	12.3 (2.2)	2.8 (0.6)	8	Yes	N/A
1900041	Mirtazapine 15–60 mg (92) Amitriptyline 50–200 mg (98)	48.6 (9.7)	65%	24.6 (3.9)	11.8 (2.3)	2.7 (0.7)	6	Yes	Outpatients
1900042	Mirtazapine 15–60 mg (77) Venlafaxine 75–375 mg (74)	44.9 (10.5)	66%	29.2 (2.9)	14.6 (1.8)	3.2 (0.6)	8	Yes	N/A

a This arm was not included in any analysis.

### Statistical analysis

All analyses were done in SAS Software 9.4. Since 25 out of the 37 studies had a duration of six weeks only, and since sex weeks of treatment should be sufficient to demonstrate an antidepressant effect, the week six visit—from which data were available in 33 out of 37 studies—was selected as primary endpoint. For analyses of early effects, week one data or—if there was no week one visit—week two data were used.

First, effect sizes—defined as the mean difference divided by the root of the variance for a particular time-point—and p-values over time were calculated for the comparison of mirtazapine vs SSRIs, venlafaxine, and amitriptyline for *i)* HDRS-17-sum, *ii)* the HDRS-6 subscale (item 1: depressed mood, 2: guilt, 7: work and activities, 8: psychomotor retardation, 10: psychic anxiety, and 13: general somatic symptoms),^10^ and *iii)* the depressed mood item. Each analysis only included trials directly comparing mirtazapine to the corresponding comparator. The analyses used a mixed model for repeated measures (MMRM) specification for the SAS PROC MIXED procedure.^11^ All models included fixed factors for trial, time (week), and treatment, as well as the interaction between time and treatment. Baseline score for the outcome parameter was included as a covariate. Within-subject correlations over time were modelled using an unstructured variance-covariance matrix and the Kenward-Roger approximation was used to approximate denominator degrees of freedom. In cases where the model did not converge using an unstructured variance-covariance matrix, an autoregressive matrix with heterogeneous variance was used.

Second, analogous MMRM models were used for the comparison of each treatment vs mirtazapine with respect to all individual HDRS-17-items at week one and week six.

Third, prompted by item-level differences in response, as well as by differing responses with respect to HDRS-17 as compared to HDRS-6 for SSRIs and mirtazapine, respectively, we conducted two follow-up analyses using the same MMRM specification. In the first of these, we excluded sleep symptoms (items 4 to 6), appetite (item 12) and weight (item 16). In the second, we excluded the aforementioned items as well as item 11 (somatic anxiety).

Fourth, indirect comparisons of amitriptyline vs SSRIs, venlafaxine, or the combination of SSRIs and venlafaxine were undertaken using the same outcome measures and model specifications as in the previous analysis; however, the fixed factor for trial was omitted since no study contributed data on both amitriptyline and SSRIs/venlafaxine. SSRIs were likewise indirectly compared with all TCAs combined. Since the inclusion of a placebo arm is known to impact the outcome of depression trials,^12^ and since placebo arms were more common in amitriptyline trials (five out of ten) than in SSRI trials (one in fourteen) and venlafaxine trials (zero out of two), trials including placebo were excluded from these analyses. Since week one data coverage was poor for the remaining studies, week two data was used to assess early differences. Moreover, since presence of psychomotor retardation at baseline was more common in the amitriptyline group than in the SSRI group—probably reflecting that amitriptyline trials were more often conducted in inpatients—the analyses of SSRIs vs amitriptyline or vs all TCAs combined were repeated after making the population more homogenous in this regard by excluding patients not displaying a score of at least two on the HDRS psychomotor retardation item (rated 0–4).

Fifth, prompted by the abovementioned difference in baseline symptomatology between trials comparing mirtazapine to amitriptyline and SSRIs, respectively, we also assessed the extent to which differences in study design may have introduced spurious between-treatment differences. To this end, we compared outcomes with mirtazapine between trials using amitriptyline, venlafaxine, or an SSRI, respectively, as comparator.

All endpoint analyses were repeated using analysis of covariance models (ANCOVA) applying the last observation carried forward (LOCF) methodology and including all patients with a post-baseline observation.

### Ethics

Since the data were anonymized and could not be traced back to the individuals once participating in the trials in question, neither obtaining informed consent nor requesting ethical permit to conduct the study was applicable. We however did request and obtain a statement from the Swedish Ethical Review Authority confirming this to be the case.

### Role of the funding source

The funders of the study had no role in study design, data collection, data analysis, data interpretation, or writing of the report. All authors had full access to the data in the study and had final responsibility for the decision to submit for publication.

## Results

### Patients

Table 1 details characteristics of the 37 included trials. The data set consisted of 5974 patients treated with placebo or an antidepressant. 381 mirtazapine patients were included in multiple analyses since they originated from three-arm studies including both placebo and an active comparator. The subpopulations thus consisted of 1578 patients (968 on mirtazapine) for mirtazapine vs placebo, 2731 patients (1362 on mirtazapine) for mirtazapine vs SSRI, 391 patients (202 on mirtazapine) for mirtazapine vs venlafaxine, 1228 patients (606 on mirtazapine) for mirtazapine vs amitriptyline, and 427 patients (218 on mirtazapine) for mirtazapine vs TCAs other than amitriptyline.

### Direct comparisons over time

**Fig. 1:**
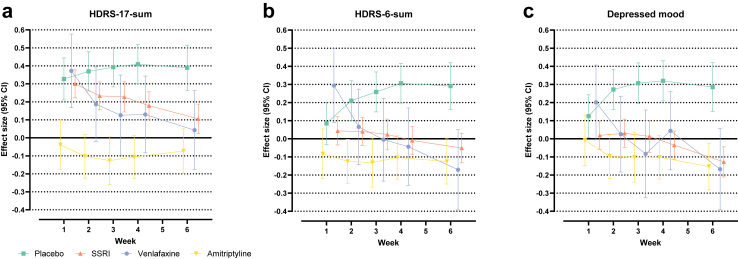
Effect sizes over time for mirtazapine vs placebo, mirtazapine vs SSRIs, mirtazapine vs venlafaxine, and mirtazapine vs amitriptyline with respect to HDRS-17-sum, HDRS-6-sum and depressed mood. **Legend:** Shown are effect sizes with 95% confidence intervals for HDRS-17-sum, HDRS-6-sum, and the depressed mood item when comparing mirtazapine with placebo, SSRIs, venlafaxine, and amitriptyline, respectively. A positive effect size suggests superiority of mirtazapine. A negative effect size suggests superiority of the comparator. If a confidence interval does not cross zero, the difference is statistically significant. a: Effect size for HDRS-17-sum, b: Effect size for HDRS-6-sum, c: Effect size for depressed mood.

Fig. 1a–c shows effect sizes (with 95% confidence intervals) vs mirtazapine for placebo, SSRIs, venlafaxine, and amitriptyline at all post-baseline timepoints with respect to HDRS-17- sum, HDRS-6-sum, and depressed mood. With respect to HDRS-17-sum, mirtazapine outperformed SSRIs throughout the trials but with attenuating effect sizes over time; the difference between mirtazapine and venlafaxine displayed a similar trajectory. With respect to HDRS-6-sum, mirtazapine never outperformed SSRIs and outperformed venlafaxine at visit one only—at endpoint, there was a non-significant tendency for both SSRIs and venlafaxine to outperform mirtazapine. With respect to depressed mood, mirtazapine never outperformed SSRIs or venlafaxine—at endpoint, patients on SSRIs (significantly) and those on venlafaxine (non-significantly) were rated lower than those on mirtazapine. Notably, considerably fewer patients were treated with venlafaxine (n = 189) than with an SSRI (n = 1369).

Throughout the study, amitriptyline numerically outperformed mirtazapine with respect to all three measures. At endpoint, this difference was significant with respect to HDRS-6-sum and depressed mood but not HDRS-17-sum. For all TCAs combined, all differences reached significance at week six (HDRS-17-sum: ES = −0.14, p = 0.01; HDRS-6-sum: ES = −0.17, p = 0.002; depressed mood: ES = −0.18, p = 0.001) (not shown in figure).

### Item-level comparisons of mirtazapine to other treatments

**Fig. 2:**
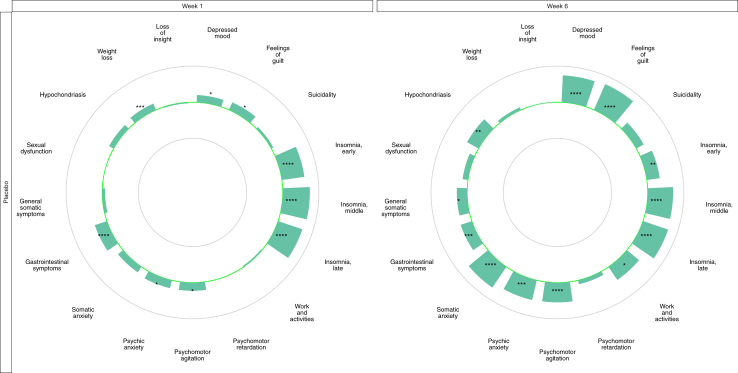
Item-wise separation for mirtazapine vs placebo ager one and six weeks of treatment. **Legend:** The green centre line represents no difference between mirtazapine and placebo. Bars pointing towards the outer circle favour mirtazapine, where a bar reaching the outer circle would correspond to a mean difference (MD) of +0.4 points. Bars pointing towards the inner circle favour placebo where a bar reaching the inner circle would correspond to a mean difference of −0.4 points. ∗ = p < 0.05, ∗∗ = p < 0.01, ∗∗∗ = p < 0.001, ∗∗∗∗ = p < 0.0001.

Mirtazapine outperformed placebo with respect to depressed mood, guilt, insomnia, psychomotor agitation, psychic anxiety, gastrointestinal symptoms, and weight loss at week one. At endpoint, mirtazapine outperformed placebo with respect to all HDRS items except suicidality, psychomotor retardation, sexual dysfunction, weight loss, and loss of insight (Fig. 2).

**Fig. 3:**
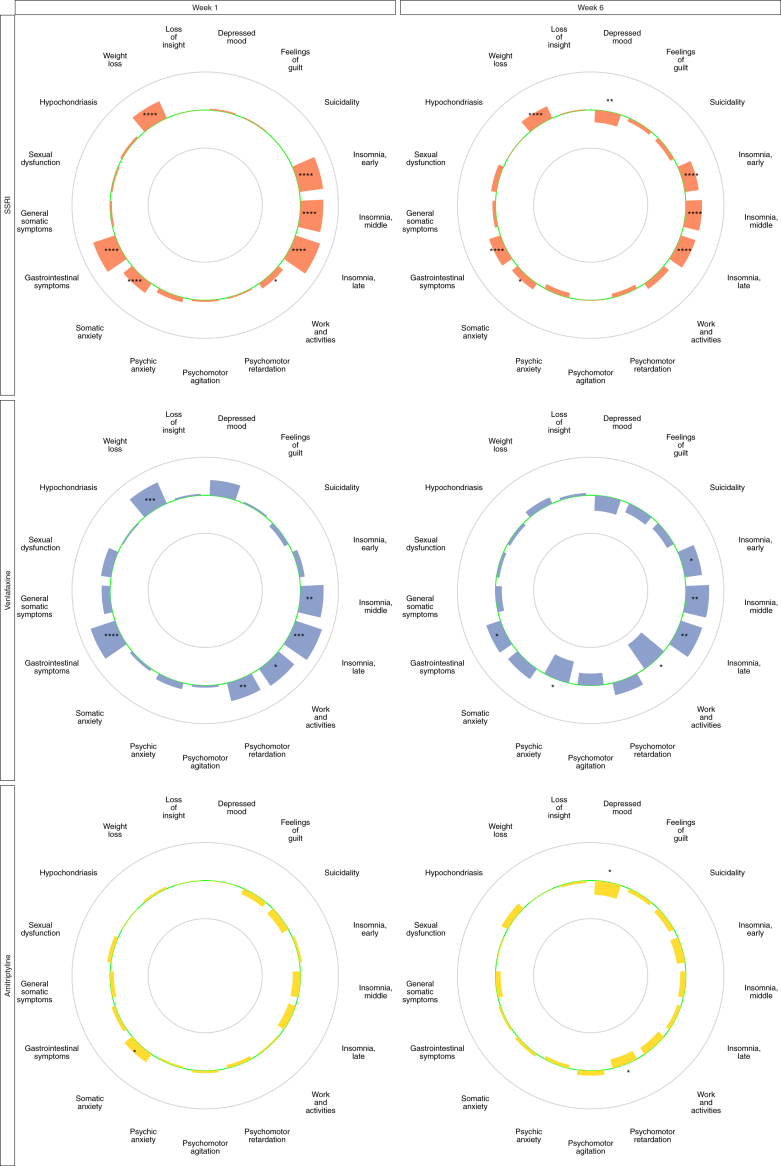
Item-wise separation for mirtazapine vs SSRIs, mirtazapine vs venlafaxine and mirtazapine vs amitriptyline, ager one and six weeks of treatment. **Legend:** The green centre line represents no difference between mirtazapine and control. Bars pointing towards the outer circle favour mirtazapine, where a bar reaching the outer circle would correspond to a mean difference (MD) of +0.4 points. Bars pointing towards the inner circle favour the active comparator where a bar reaching the inner circle would correspond to a mean difference of −0.4 points. ∗ = p < 0.05, ∗∗ = p < 0.01, ∗∗∗ = p < 0.001, ∗∗∗∗ = p < 0.0001.

Mirtazapine outperformed SSRIs with respect to sleep items, work and activities, somatic anxiety, gastrointestinal symptoms, and weight loss at week one, and a similar pattern was observed also for the mirtazapine vs venlafaxine comparison. In contrast, at week six both SSRIs and venlafaxine performed numerically better than mirtazapine on several affective and cognitive symptoms of depression. SSRI treatment led to a significantly larger decrease in depressed mood and venlafaxine treatment did the same for psychic anxiety and work and activities (Fig. 3). When compared to a combined SSRI and SNRI group, mirtazapine showed less efficacy at endpoint with respect to reduction in depressed mood, psychic anxiety, and suicidality (Supplementary Figure S1). In contrast, mirtazapine outperformed SSRIs, venlafaxine, and the combined SSRI/SNRI group with respect to sleep items, somatic anxiety, gastrointestinal symptoms, and weight loss (Fig. 3 & Supplementary Figure S1).

At week one, the only significant difference between mirtazapine and amitriptyline was that somatic anxiety was rated higher in the amitriptyline group. At week six, amitriptyline outperformed mirtazapine with respect to reduction in depressed mood and psychomotor retardation (Fig. 3). As a group, all tricyclics combined outperformed mirtazapine with respect to depressed mood, guilt, suicidality, early and middle insomnia, work and activities, and psychomotor retardation (Supplementary Figure S2).

### Indirect comparisons

**Fig. 4:**
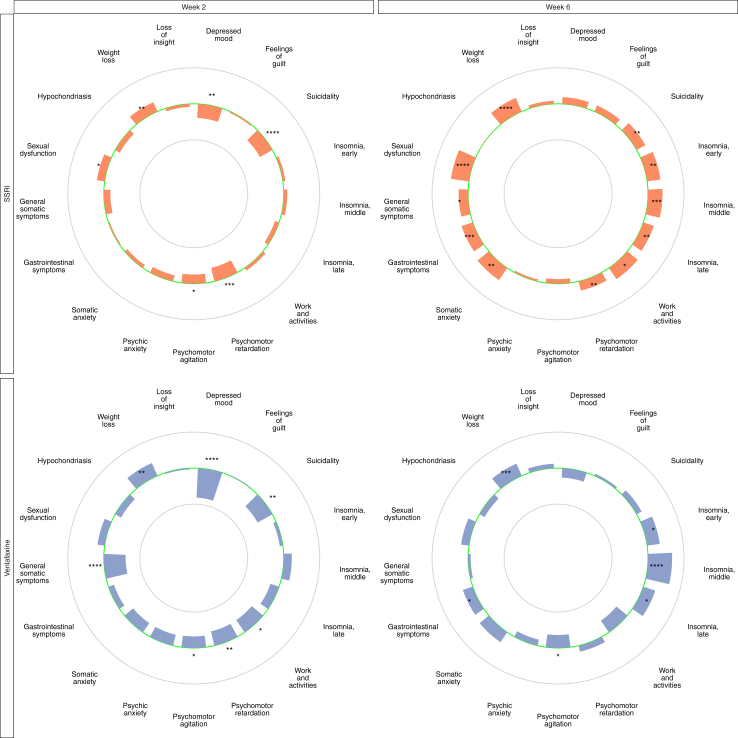
Item-wise separation for amitriptyline vs SSRIs and amitriptyline vs venlafaxine ager two and six weeks of treatment. **Legend:** The green centre line represents no difference between amitriptyline and the SSRIs and venlafaxine, respectively. Bars pointing towards the outer circle favour amitriptyline, where a bar reaching the outer circle would correspond to a mean difference (MD) of +0.4 points. Bars pointing towards the inner circle favour SSRIs and venlafaxine, respectively, where a bar reaching the inner circle would correspond to a mean difference (MD) of +0.4 points. ∗ = p < 0.05, ∗∗ = p < 0.01, ∗∗∗ = p < 0.001, ∗∗∗∗ = p < 0.0001.

An indirect comparison of amitriptyline vs SSRIs found amitriptyline to outperform SSRIs at endpoint with respect to HDRS-17-sum (ES 0.28; p < 0.0001), HDRS-6-sum (ES 0.13, p = 0.044) and the following individual symptoms: suicidality, all three insomnia items, work and activities, psychomotor retardation, somatic anxiety, gastrointestinal symptoms, general somatic symptoms, sexual dysfunction, and weight loss (Fig. 4). An indirect comparison of SSRIs vs all TCAs combined resulted in similar outcomes (Supplementary Figure S3). Comparing venlafaxine with amitriptyline revealed fewer significant differences—while amitriptyline-treated patients rated lower with respect to all insomnia items, the gastrointestinal symptom item, and the weight loss item, venlafaxine outperformed amitriptyline with respect to reduction in psychomotor agitation (Fig. 4).

When interpreting the indirect comparisons, it should be noted that design and patient characteristics in trials with one comparator may differ systematically from those with another comparator and that this could impact outcomes. In this vein, baseline rating of, e.g., psychomotor retardation, which is a key feature of melancholic depression, was higher in amitriptyline trials than in SSRI trials (Supplementary Table S2) which is in line with the former more often having been undertaken in inpatients (Table 1). Notably, the trajectory of response to mirtazapine in trials with different comparators indeed differed markedly (Supplementary Figure S4) usually following the trajectory of the comparator (not shown). However, endpoint outcomes were largely the same for mirtazapine regardless of comparator.

To assess if differences with respect to prevalence of psychomotor retardation in the different trial populations could explain the difference observed between amitriptyline and SSRIs, the indirect comparisons were repeated after exclusion of patients rated less than two with respect to this item at baseline (Supplementary Figure S5). At endpoint, amitriptyline again outperformed SSRIs with respect to HDRS-17-sum (ES: 0.32, p = 0.0005) and HDRS-6-sum ES 0.26; p = 0.0044) and also with respect to, e.g., depressed mood, guilt, suicidality, work and activities, psychomotor retardation, and lack of insight.

### Sensitivity and follow-up analyses

The outcomes of analyses using LOCF did not materially differ from those obtained with MMRM (Supplementary Figure S6). Analyses contrasting mirtazapine to SSRIs using an abbreviated HDRS-17 that excluded items 4 to 6 (sleep), item 12 (appetite) and item 16 (weight) found non-significant differences at all follow-up times (week 1–6; ES-range: −0.02 to 0.07; p-range: 0.10 to 0.70). Analyses excluding also item 11 (somatic anxiety) yielded similar results (ES-range −0.03 to 0.05; p-range: 0.26 to 0.94).

## Discussion

The main question addressed in this study was to what extent previous data suggesting amitriptyline to be superior to other antidepressants, and mirtazapine to be at least as effective as the more commonly used SSRIs and SNRIs,^1^ can be explained by differences between these drugs vs the SSRIs/SNRIs with respect to how they impact sleep and appetite. While mirtazapine outperformed SSRIs (but not venlafaxine) with respect to the conventional effect parameter, i.e., the HDRS-17-sum, this was entirely explained by the former drug being superior with respect to sleep items and items reflecting appetite and gastrointestinal dysfunction. Though clearly superior to placebo with respect to most HDRS items (Fig. 2), mirtazapine was thus inferior to SSRIs in reducing depressed mood, inferior to venlafaxine in reducing psychic anxiety and work and activities, and inferior to the combined SSRI plus SNRI group in reducing depressed mood, psychic anxiety, and suicidality (Fig. 3 and Supplementary Figure S1). Due to its potent sedative and orexigenic effects, mirtazapine however still outperformed SSRIs on the HDRS-17-sum after six weeks of treatment (Fig. 1). Conversely, amitriptyline—and more clearly the pooled TCA group—performed as well or better than mirtazapine on all individual HDRS items (Fig. 3, Supplementary Figure S2).

The observation that the sedative effect of mirtazapine impacts its apparent antidepressant efficacy as reflected by a reduction in HDRS-17-sum is in line with previous reports.^13^ The present study shows that also its well-established orexigenic effect, probably caused by H1 and 5-HT2C antagonism, also contributes to mirtazapine appearing more efficacious than antidepressants more inclined to exert the opposite effect, i.e., the SSRIs and SNRIs.^14^^,^^15^ Notably, the gastrointestinal symptoms item of the HDRS is mainly used to rate appetite.^4^

One might expect a H1-blocking sedative antidepressant, such as mirtazapine, not only to cause a rapid improvement in sleep, as here confirmed, but also to produce an early non-specific anxiety-reducing effect. There was, however, no difference between mirtazapine and SSRIs with respect to the rating of psychic anxiety during the first weeks of treatment. With respect to somatic anxiety, however, patients on SSRIs displayed higher ratings than those on mirtazapine already at the first treatment visit, which is in line with previous studies showing SSRIs to exert a slight and transient exacerbation of this item during the first week also as compared to placebo.^16^ The difference between SSRIs and mirtazapine with respect to the somatic anxiety item however persisted until endpoint, hence contributing to the superiority of the latter with respect to reduction in HDRS-17-sum. Of note is that the somatic anxiety item in HDRS captures, e.g., gastrointestinal problems; this difference may hence tentatively be explained by gastrointestinal dysfunction being a common side effect with SSRIs/SNRIs,^14^ but less so with mirtazapine, which has even been attributed a beneficial impact in conditions of gastrointestinal dysfunction (tentatively due to its 5-HT3-blocking property).^17^ Also patients on amitriptyline (and tricyclics combined) displayed higher early rating with respect to somatic anxiety after one week of treatment, which is in line with these drugs also being SERT blockers.

Whereas it has often been claimed that SSRIs, to a greater extent than non-serotonergic antidepressants, may provoke suicidal ideation,^18^ especially shortly after the onset of treatment, previous meta-analyses comparing SSRIs with placebo have shown that SSRIs on average reduce suicidality already 1–2 weeks after onset of treatment (and onwards).^19^, ^20^, ^21^, ^22^ In the same vein, the present study provides no support for SSRIs being more likely to enhance suicidality than mirtazapine—on the contrary, ratings of suicidality numerically favoured both SSRIs and venlafaxine over mirtazapine. This difference reaching significance when the SSRI- and venlafaxine-treated populations were combined (Supplementary Figure S1).

In line with some early trials suggesting mirtazapine to be associated with a more rapid onset of action than the SSRIs,^23^, ^24^, ^25^ a separation favouring mirtazapine with respect to HDRS-17-sum over SSRIs or venlafaxine was found already after one week of treatment. This superiority, however, was also entirely explained by an early difference with respect to items reflecting sleep, weight, and gastrointestinal symptoms (Fig. 3, Supplementary Figure S1).

When introduced, mirtazapine was often claimed to be as effective as tricyclic antidepressants in severely depressed inpatients,^26^ but there has also been trials casting doubt on this notion.^27^ The present item-level analysis found amitriptyline to be numerically superior to mirtazapine with respect to most items at endpoint, and significantly so with respect to HDRS-6-sum, depressed mood, and psychomotor retardation. Moreover, the combined TCA group outperformed mirtazapine also with respect to the guilt, suicidality, and work and activities items. This study hence suggests tricyclics to be superior to mirtazapine with respect to antidepressant efficacy, and not least with respect to symptoms typical of melancholic depression.

The relative efficacy of tricyclics such as amitriptyline vis-à-vis the SSRIs has also been a long-standing issue of debate, some studies suggesting that the SSRIs may be somewhat inferior to tricyclics.^28^, ^29^, ^30^, ^31^ The present mirtazapine-focused dataset did not include any head-to-head comparisons between amitriptyline and an SSRI, and hence cannot provide any definite conclusions regarding the relative efficacy of the two. With this caveat borne in mind, data from trials comparing amitriptyline with SSRIs were nevertheless pooled to allow for indirect comparisons. These showed SSRIs to be numerically inferior to amitriptyline with respect to almost all items at endpoint, and significantly so with respect to symptoms typical of melancholic depression, such as depressed mood, psychomotor retardation, suicidality, and inability to work.

While data on setting was unavailable for many SSRI trials, those with known setting were almost entirely conducted in outpatients whereas four out of ten amitriptyline trials were conducted in inpatients. In line with this, psychomotor retardation was markedly less prevalent at baseline in SSRI trials than in amitriptyline trials. Illustrating the possible importance of factors related to trial design for the outcome of depression trials, there were notable differences in the trajectories of the antidepressant effect of mirtazapine in trials vs amitriptyline as compared to those vs SSRIs or vs venlafaxine. This observation may have implications for the wider meta-analytical field, where including not only head-to-head studies but also indirect comparisons has increasingly become the norm.^1^

To make the populations more homogenous with respect to presence of melancholic features, indirect comparison of amitriptyline vs SSRIs or venlafaxine including only patients with at least 2 points on the psychomotor retardation item at baseline were undertaken—though many symptoms still displayed significant baseline differences, this manoeuvre did reduce the difference in baseline rating of psychomotor retardation. Also in this subpopulation, amitriptyline outperformed SSRIs with respect to this item, as well as with respect to lack of insight and guilt. While these results are compatible with the notion that tricyclics are indeed more efficacious than SSRIs, not least in patients with melancholic features, they should, as underlined, be interpreted with due caution since they are indirect.

Amitriptyline and other tricyclic antidepressants are marred by overdose toxicity and certain tolerability issues, including anticholinergic side effects which may be particularly problematic in the elderly—that they are no longer regarded as first line treatment for depression is hence justified. However, their apparent superiority in terms of efficacy does suggest that they may still have a role to play—especially in difficult-to-treat cases of melancholic depression—and warrants further research into why they display this superiority. While in this material mirtazapine's clear superiority over SSRIs/SNRIs in promoting sleep and appetite did not translate into better antidepressant efficacy as measured by cognitive and affective symptoms of depression, mirtazapine could still hold an advantage in patients for whom sleep dysfunction and reduced appetite are major complaints.

This study has several limitations. First, we did not consider the doses given; this may be of particular relevance for amitriptyline where doses lower than those usually regarded as optimal in terms of efficacy, e.g., 150 mg/day, have often been applied in trials, or the fact that the relatively slow dose escalation of tricyclics might impact when they are compared to drugs with a faster (or no) gradual increase in dosing.^32^ Second, differential dropout between treatments may have impacted outcomes. We used MMRM analyses to partly compensate for this since they are better at handling missing data than LOCF-ANCOVA under most scenarios.^11^ Such adjustments are, however, not perfect and it is possible that differences, e.g., in the propensity for early side effects may have impacted certain treatments unfavourably. Third, we lacked information with respect to many of the trials including aspects such as inclusion criteria, inpatients/outpatient setting, somatic and psychiatric comorbidities, where and when the study was performed etc; hence, we could not address the possible impact of such aspects on the outcome. This is of particular importance when interpreting the indirect comparisons.

In conclusion, the present study illustrates the importance of symptom specific analyses when assessing antidepressant efficacy. The superiority of mirtazapine vis-à-vis SSRIs in reducing HDRS-17-sum (Fig. 1) was entirely explained by its impact on sleep and appetite, and lack of gastrointestinal side effects (Fig. 3). With respect to reducing affective and cognitive symptoms of depression, SSRIs and venlafaxine were never inferior—and sometimes superior—to mirtazapine after six weeks of treatment. While the sedative and orexigenic properties of amitriptyline gives it a similar advantage compared to SSRIs and SNRIs when efficacy is measured by HDRS-17-sum, amitriptyline was as or more effective than mirtazapine across the board, and indirect analyses comparing SSRIs to amitriptyline were indicative of the latter possibly being more efficacious also as compared to these with regard to the affective and cognitive symptoms of depression.

## Contributors

All authors designed the study. AL and EE acquired the data. FH and AL verified the data and conducted all analyses. FH drafted the manuscript. All authors critically reviewed the manuscript, approved its submission, and accept responsibility for its content.

## Data sharing statement

Researchers can apply to obtain the data used in this publication by submitting a request through the EngageZone portal (Merck Sharp & Dohme). The SAS code used to produce the results, and the R code used to produce the illustrations, is available from the authors on request.

## Declaration of interests

FH has received speaker fees from Janssen. EE has been on advisory boards and/or received speaker's honoraria and/or research grants from H Lundbeck and Janssen. AL declares no competing interests.

## Appendix A Supplementary data

Model results
