# Supplementary material for: Impact of sedative and appetite-increasing properties on the apparent antidepressant efficacy of mirtazapine, selective serotonin reuptake inhibitors and amitriptyline: an item-based, patient-level meta-analysis
Source: eClinicalMedicine. 2024 Nov 7;77:102904. doi: 10.1016/j.eclinm.2024.102904 (PMC11576391; doi:10.1016/j.eclinm.2024.102904)
Supplement: Supplementary Figures and Tables [file mmc2.pdf]

Supplementary information for

Impact of sedative and appetite-increasing properties on the apparent antidepressant efficacy of mirtazapine, selective serotonin reuptake inhibitors and amitriptyline: an item-based, patient-level meta-analysis

Fredrik Hieronymus, Alexander Lisinski, Elias Eriksson

Table of contents

Supplementary table 1. List of requested trials
Supplementary table 2. Item scores at baseline for SSRIs, venlafaxine, and amitriptyline
Supplementary figure 1. Item-wise separation between mirtazapine and SSRI+Venlafaxine after one and six weeks of treatment
Supplementary figure 2. Item-wise separation between mirtazapine and TCAs after one and six weeks of treatment
Supplementary figure 3. Item-wise separation for TCAs vs SSRIs and TCAs vs venlafaxine after two and six weeks of treatment
Supplementary figure 4. Differences in the response to mirtazapine between trials comparing mirtazapine to amitriptyline and trials comparing mirtazapine to SSRIs
Supplementary figure 5. Item-wise separation for TCAs vs SSRIs and TCAs vs venlafaxine after two and six weeks of treatment in patients with a score of at least two on psychomotor retardation
Supplementary figure 6. Item-wise analyses of endpoint (week 6) differences using last observation carried forward-methodology

Supplementary table 1. List of requested trials.

Included trials

**Trial identifie r**	**Description**	**Matching publication**
003-002	The effectiveness of 6-azamianserin (Org 3770) in depressed outpatients.	J Affect Disord. 1995; **34**: 165-71
003-003	A placebo-controlled study of Org 3770 in moderately depressed outpatients.	Not identified
003-008	A controlled dose range study of Org 3770 in outpatients with major depression.	Not identified
003-020	A controlled study of Org 3770 in outpatients with major depression.	Not identified
003-021	A controlled study of Org 3770 in out-patients with major depression.	Not identified
003-022	A double-blind comparison of Org 3770, amitriptyline, and placebo in major depression.	J Clin Psychiatry. 1995; **56**: 519-25
003-023	Org 3770 (mirtazapine) versus trazodone: A placebo controlled trial in depressed elderly patients.	Halikas et. al. Human Psychopharmacology: Clinical and Experimental, 1995;**10**(Suppl 2):125–133
003-024	Mirtazapine vs. amitriptyline vs. placebo in the treatment of major depressive disorder.	Smith et. al. Psychopharmacol Bull. 1990; **26**: 191-6
003-042	An eight-week, multicenter, double-blind placebo-controlled fixed dose response study to define the antidepressant effectiveness and sedation properties of Remeron in outpatients with major depression.	Not identified
003-048	Multi-center, randomized, double-blind, fluoxetine and placebo-controlled study of the efficacy and safety of RemeronSolTab orally disintegrating tablets (mirtazapine ODT) in subjects with major depressive disorder.	Not identified
003-900	Multicenter, randomized, double-blind, sertraline-controlled study of the efficacy and safety of Remeron (mirtazapine) in subjects with major depressive disorder who failed on SSRI treatment due to lack of efficacy.	Not identified
003-901	Mirtazapine vs. Paroxetine Study G. Double-blind, randomized comparison of mirtazapine and paroxetine in elderly depressed patients.	Schatzberg et. al. Am J Geriatr Psychiatry. 2002; **10**: 541-50
22521	A double-blind, randomized, group-comparative study of the tolerability and efficacy of 6 weeks' treatment with mirtazapine or fluoxetine in depressed Chinese patients.	Hong et. al. J Clin Psychiatry. 2003; **64**: 921-6
22532	A double-blind, randomized, flexible dose, fluvoxamine-controlled, group- comparative trial, comparing the efficacy and safety of six weeks treatment with Org 3770 and fluvoxamine in patients suffering from major depressive disorder (according to DSM-IV).	Not identified
83047	A double-blind, amitriptyline-controlled, rising dose efficacy and safety study with Org 3770 administered orally for a period of six weeks to hospitalized patients with major depressive illness (multicentre study).	Not identified
84023	Double-blind study of mirtazapine and placebo in hospitalized patients with major depression.	Vartiainen et. al. Eur Neuropsychopharmacol. 1994; **4**: 145-50

84050	A double-blind, placebo-controlled efficacy and safety study with Org 3770 (15, 30 and 60 mg daily) administered orally for a period of 5 weeks to patients suffering from major depression.	Not identified
84062	A single-centre, double-blind, randomized group comparative study to evaluate the effect of six weeks treatment with Org 3770 and amitriptyline administered to patients with major depressive disorder in comparison with the effect of placebo.	Not identified
84072	A double-blind, clomipramine-controlled, rising dose efficacy and safety study with Org 3770 administered orally for a period of six weeks to patients suffering fom major depression (multicentre study).	Not identified
85003	A double-blind, doxepin-controlled, flexible dose efficacy and safety study with Org 3770 administered orally for a period of six weeks to patients with major depressive episode (multicenter study).	Marttila et. al. Eur Neuropsychopharmacol. 1995; **5**: 441-6
85027	A randomised, double-blind, placebo-controlled, 5-weeks' study of Org 3770 (mirtazapine) in major depression.	Khan et. al. Human Psychopharmacology: Clinical and Experimental. 1995; **10**(Suppl 2):119–124
85042	A double-blind, amitriptyline-controlled, flexible dose efficacy and safety study with Org 3770 administered orally for a period of five weeks to patients with major depressive episode (multicentre study).	Not identified
85146	A double-blind, randomized, group-comparative trial to assess the relative efficacy of Org 3770 in the acute treatment of major depression in comparison to amitriptiline (multicenter study).	Not identified
86017	A single-centre, double-blind, randomized group- comparative study of the differential antidepressant effects of Org 3770 and imipramine in depressed inpatients.	Not identified
88013	A double-blind multicentre comparison of mirtazapine and amitriptyline in elderly depressed patients.	Hoyberg et. al. Acta Psychiatr Scand. 1996; **93**: 184-90
C-1763	Mirtazapine orally disintegrating tablets versus venlafaxine extended release: a doubleblind, randomized multicenter trial comparing the onset of antidepressant response in patients with major depressive disorder.	Benkert et. al. Journal of Clinical Psychopharmacology 2006; **26**: 75-78
E-1527	A multicenter, randomized, double-blind group comparative study comparing the tolerability and efficacy of 6 weeks treatment with Remeron (Org 3770) and fluoxetine in several depressed patients.	Wheatley et. al. J Clin Psychiatry. 1998; **59**: 306-12.
E-1559	Mirtazapine compared with paroxetine in major depression	Benkert et. al. J Clin Psychiatry. 2000; **61**: 656-63
E-1563	A multi-center, randomized, two-way, double- blind, long-term study to prove the equivalence of the efficacy of Org 3770 (mirtazapine) and amitriptyline after six weeks of treatment and to compare the efficacy and tolerability of these two substances over a maximum period of six months in subjects with a major depression (according to DSM-III-R criteria).	Not identified
E-1565	Protocol for a single centre, randomized double- blind group comparative study comparing the efficacy, tolerability and cardiac safety of 6 weeks treatment with Mirtazapine (Remeron) and Paroxetine (Aropax) in depressed subjects.	Not identified
E-1569	A multicentre, randomised, double-blind group comparative study comparing the tolerability of six weeks treatment with Remeron (Org 3770) and paroxetine in depressed patients in general practice.	Not identified
E-1620	Comparison of the effects of mirtazapine and fluoxetine in severely depressed patients.	Versiani et. al. CNS Drugs. 2005; **19**: 137-46

E-1621	A multicenter, double blind, randomized, venlafaxine controlled efficacy, safety and tolerability study (phase IIIb/phase IV) with mirtazapine (Org 3770) in severely depressed patients with melancholic features.	Guelfi et. al. J Clin Psychopharmacol. 2001; **21** :425-31.
E-1639	A single-center, randomized, double blind group comparative study on the therapeutic effects of six weeks treatment with mirtazapine, paroxetine and their combination in 60 patients with major depression.	Not identified
E-1690	Mirtazapine orally disintegrating tablet versus sertraline: a prospective onset of action study.	Behnke et. al. J Clin Psychopharmacol. 2003; **23** :358-64
E-1721	A randomized, double-blind, 24-week study comparing the efficacy and tolerability of mirtazapine and paroxetine in depressed patients in primary care.	Wade et. al. Int Clin Psychopharmacol. 2003; **18**: 133-41
P9902	A double-blind, randomized, flexible dose, fluvoxamine-controlled, group- comparative trial, comparing the efficacy and safety of six weeks treatment with Org 3770 and fluvoxamine in patients suffering from major depressive disorder (according to DSM-IV).	Not identified

Excluded trials

Trial identifier	Reason for exclusion	Description	Matching publication
82028	No HDRS	A phase II, double-blind, diazepam- and amitriptyline-controlled, rising dose efficacy and safety study with Org3770 administred orally for a period of 4 weeks to patients suffering from depression (multicenter study).	Not identified
85004	No relevant comparator	A double-blind, maprotiline-controlled, flexible dose efficacy and safety study with Org 3770 administered orally for a period of six weeks to hospitalized patients with major depressive episode (multicenter study).	Not identified
85004-2	No relevant comparator	A double-blind, randomized group comparative study to evaluate the effect of six weeks treatment with Org 3770 and maprotiline administered to patients with major depressive episode.	Not identified
85031	No relevant comparator	Mirtazapine is more effective than trazodone: a double-blind controlled study in hospitalized patients with major depression.	Moffaert et. al. Int Clin Psychopharmacol. 1995 ;**10**: 3-9
E-1562	No HDRS	Efficacy and tolerability of mirtazapine versus citalopram: a double-blind, randomized study in patients with major depressive disorder.	Leinonen et. al. Int Clin Psychopharmacol. 1999; **14**: 329-37
E-1659	Same as E-1569		
E-1627	Open label	A Single Center, Open, Randomized, Maprotiline Controlled Study Versus Remeron (Org 3770) in Depressed Patients.	Not identified
36801	Not identified		
Honig 2007	No data (academic trial)	Treatment of post-myocardial infarction depressive disorder: a randomized, placebo-controlled trial with mirtazapine.	Honig et. al. Psychosom Med. 2007; **69**: 606-13.

Kinoshita 2009	No data (academic trial)	Double-blind placebo controlled study of mirtazapine, a novel antidepressant, in Japanese patients with depression or depressive state.	Kinoshita et. al. Jpn J Clin Psychopharmacol 2009; **12**: 289-306.
Mullin 1996	No data (academic trial)	A multicentre, double-blind, amitriptyline-controlled study of mirtazapine in patients with major depression.	Mullin et. al. J Psychopharmacol. 1996; **10**: 235-40.
Wheatley 1998	Same as E-1527		

Supplementary table 2. Item scores at baseline for SSRIs, venlafaxine, and amitriptyline

	Full population			Psychomotor retardation >= 2		
**Item**	**SSRI; mean (SD)**	**Venlafaxine; mean (SE)**	**Amitriptyline; mean (SE)**	**SSRI; mean (SE)**	**Venlafaxine; mean (SE)**	**Amitriptyline; mean (SE)**
**Depressed mood**	2·9 (0·69)	3·0 (0·63)	2·7 (0·62)	3·0 (0·62)	3·2 (0·60)	2·9 (0·61)
**Feelings of guilt**	1·5 (0·82)	1·8 (0·90)	1·4 (0·88)	1·6 (0·83)	2·1 (0·98)	1·5 (0·90)
**Suicidality**	1·0 (0·87)	1·2 (0·89)	1·2 (0·91)	1·1 (0·87)	1·4 (0·89)	1·3 (0·98)
**Insomnia. early**	1·3 (0·81)	1·5 (0·74)	1·5 (0·70)	1·4 (0·80)	1·4 (0·74)	1·5 (0·68)
**Insomnia. middle**	1·4 (0·71)	1·4 (0·73)	1·4 (0·68)	1·4 (0·69)	1·4 (0·70)	1·4 (0·72)
**Insomnia. late**	1·3 (0·80)	1·4 (0·78)	1·6 (0·62)	1·2 (0·80)	1·3 (0·80)	1·6 (0·60)
**Work and activities**	2·8 (0·78)	3.0 (0·64)	2·7 (0·79)	3·0 (0·75)	3·1 (0·58)	3·0 (0·75)
**Psychomotor retardation**	1·1 (0·89)	1·2 (0·99)	1·4 (0·97)	2·2 (0·39)	2·3 (0·46)	2·3 (0·46)
**Psychomotor agitation**	1·1 (0·97)	1·2 (0·97)	1·0 (0·92)	1·2 (1·01)	1·1 (1·03)	0·7 (0·86)
**Psychic anxiety**	2·4 (0·86)	2·4 (0·74)	2·2 (0·83)	2·4 (0·86)	2·5 (0·63)	2·2 (0·88)
**Somatic anxiety**	1·9 (0·89)	2·2 (0·72)	1·9 (0·85)	1·9 (0·91)	2·2 (0·77)	1·8 (0·88)
**Gastrointestinal symptoms**	0·8 (0·73)	0·9 (0·73)	1·1 (0·69)	1·0 (0·74)	1·1 (0·72)	1·2 (0·65)
**General somatic symptoms**	1·6 (0·56)	1·6 (0·54)	1·5 (0·61)	1·5 (0·57)	1·5 (0·58)	1·5 (0·61)
**Sexual dysfunction**	1·3 (0·75)	1·5 (0·70)	1·1 (0·84)	1·4 (0·71)	1·4 (0·75)	1·1 (0·86)
**Hypochondriasis**	1·1 (1·01)	1·3 (0·96)	1·2 (1·01)	1·3 (1·04)	1·4 (0·98)	1·1 (1·04)
**Weight loss**	0·5 (0·77)	0·5 (0·81)	0·7 (0·79)	0·5 (0·79)	0·4 (0·73)	0·7 (0·81)
**Loss of insight**	0·3 (0·51)	0·3 (0·51)	0·3 (0·56)	0·4 (0·57)	0·4 (0·61)	0·4 (0·60)

Supplementary figure 1. Item-wise separation between mirtazapine and SSRI+Venlafaxine after one and six weeks of treatment

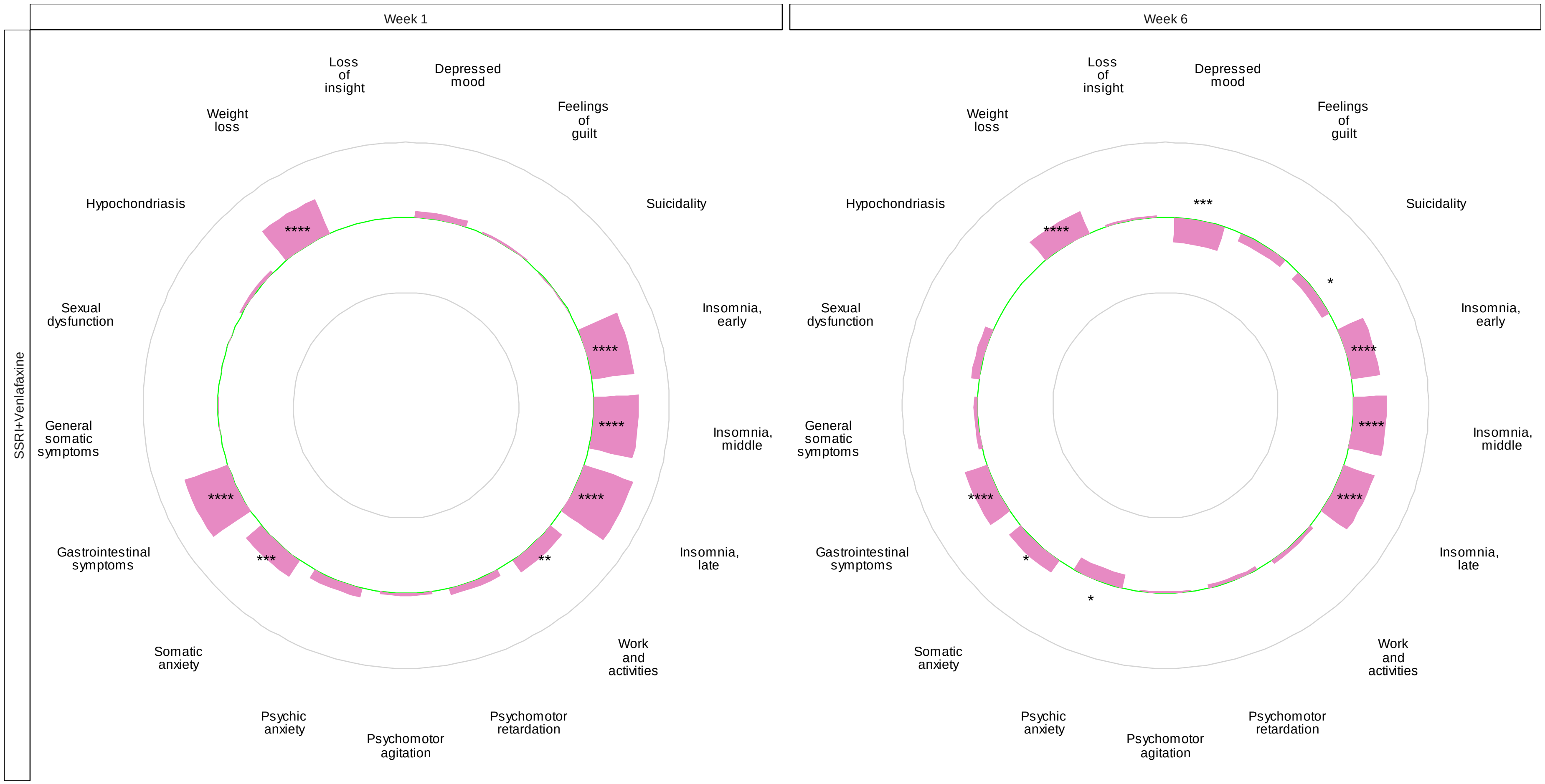

The green centre line represents no difference between mirtazapine and control. Bars pointing towards the outer circle favour mirtazapine, where a bar reaching the outer circle would correspond to a mean difference (MD) of +0·4 points. Bars pointing towards the inner circle favour the SSRI/SNRI group where a bar reaching the inner circle would correspond to a mean difference of -0·4 points. * = p < ·05, ** = p < ·01, *** = p < ·001, **** = p < ·0001.

Supplementary figure 2. Item-wise separation between mirtazapine and TCAs after one and six weeks of treatment

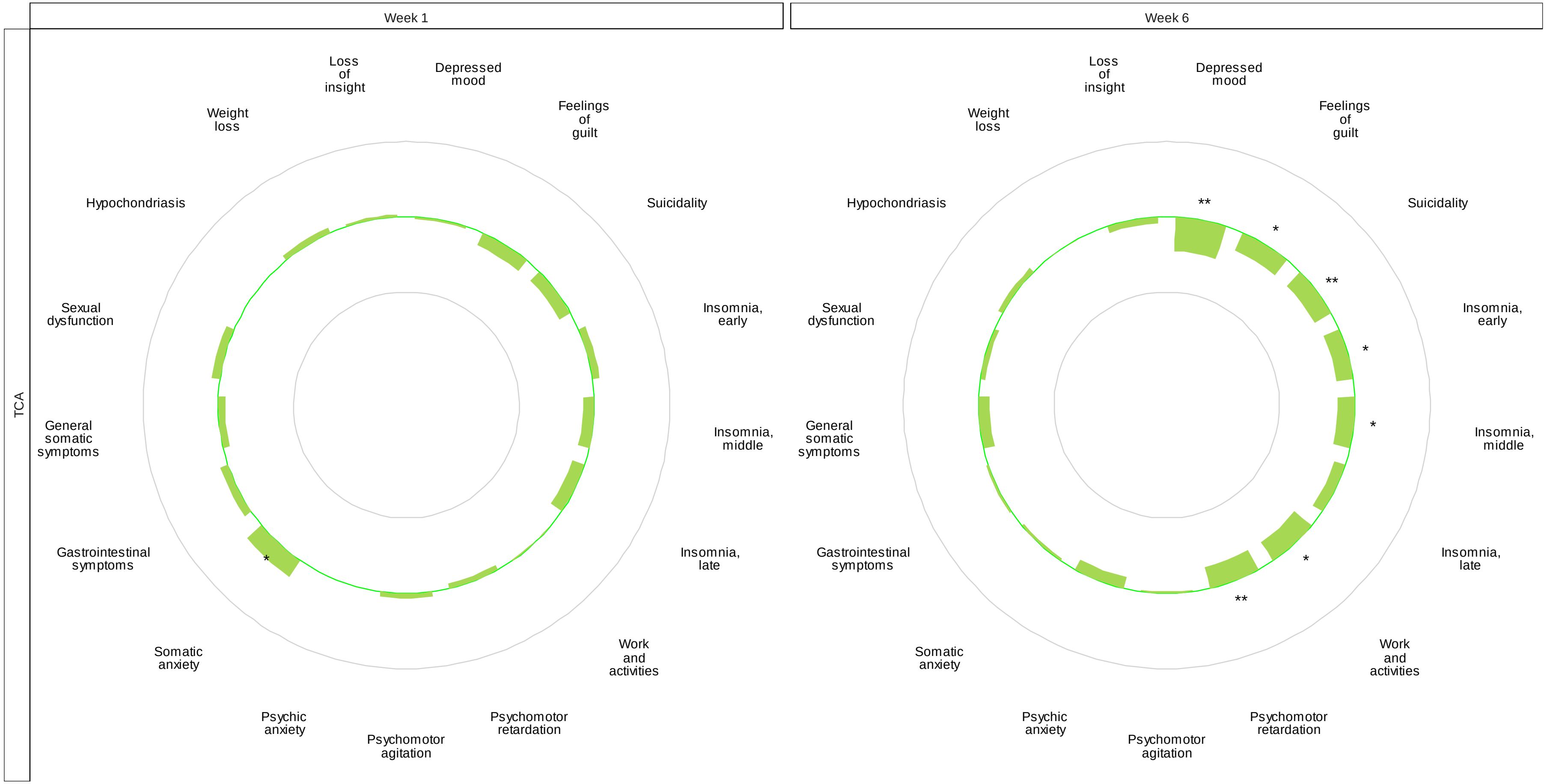

The green centre line represents no difference between mirtazapine and control. Bars pointing towards the outer circle favour mirtazapine, where a bar reaching the outer circle would correspond to a mean difference (MD) of +0·4 points. Bars pointing towards the inner circle favour the tricyclics where a bar reaching the inner circle would correspond to a mean difference of -0·4 points. * = p < ·05, ** = p < ·01, *** = p < ·001, **** = p < ·0001.

Supplementary figure 3. Item-wise separation for TCAs vs SSRIs and TCAs vs venlafaxine after two and six weeks of treatment

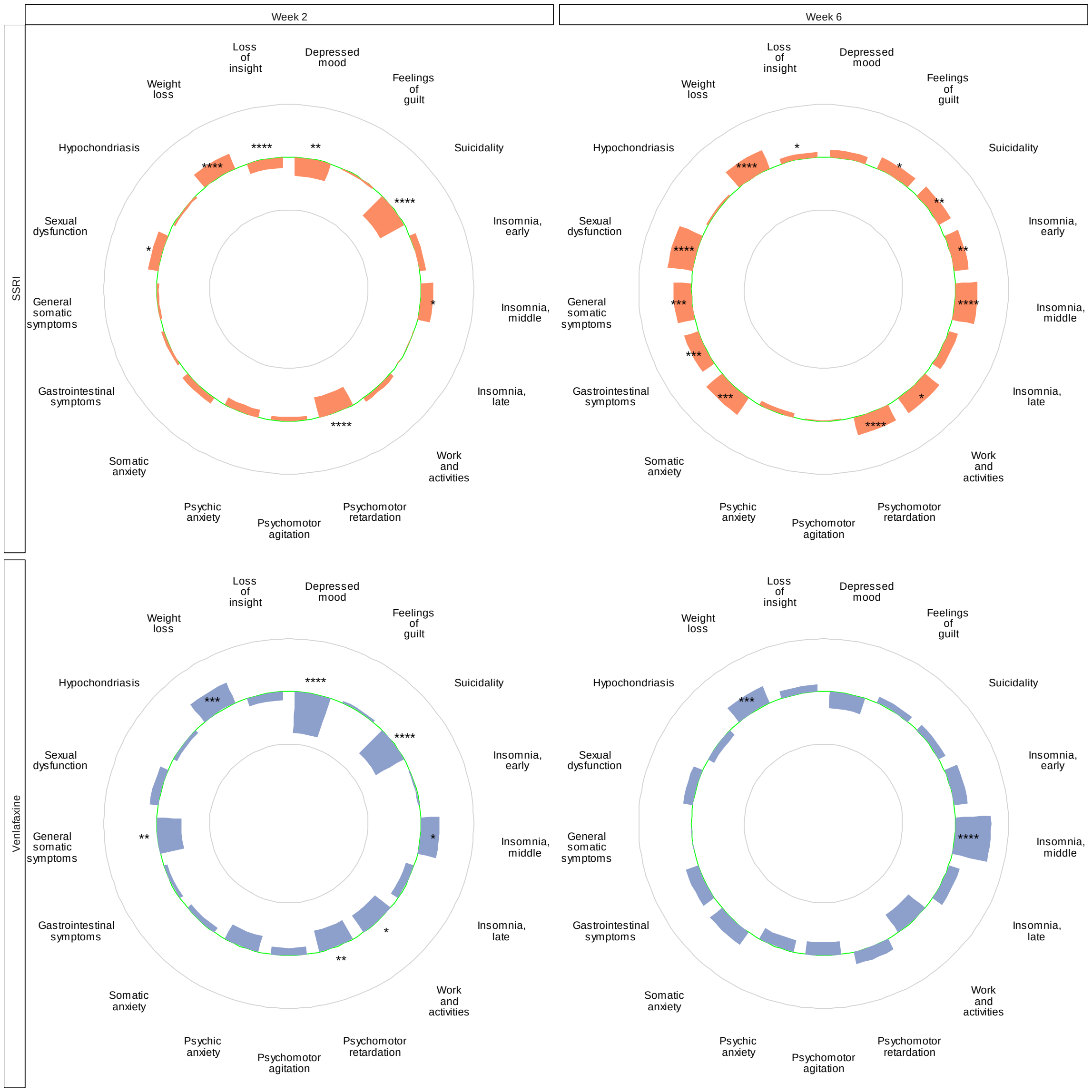

The green centre line represents no difference between mirtazapine and control. Bars pointing towards the outer circle favour the tricyclics, where a bar reaching the outer circle would correspond to a mean difference (MD) of +0·4 points. Bars pointing towards the inner circle favour the SSRI and venlafaxine, respectively, where a bar reaching the inner circle would correspond to a mean difference of -0·4 points. * = p < ·05, ** = p < ·01, *** = p < ·001, **** = p < ·0001.

Supplementary figure 4. Differences in the response to mirtazapine between trials comparing mirtazapine to amitriptyline and trials comparing mirtazapine to SSRIs

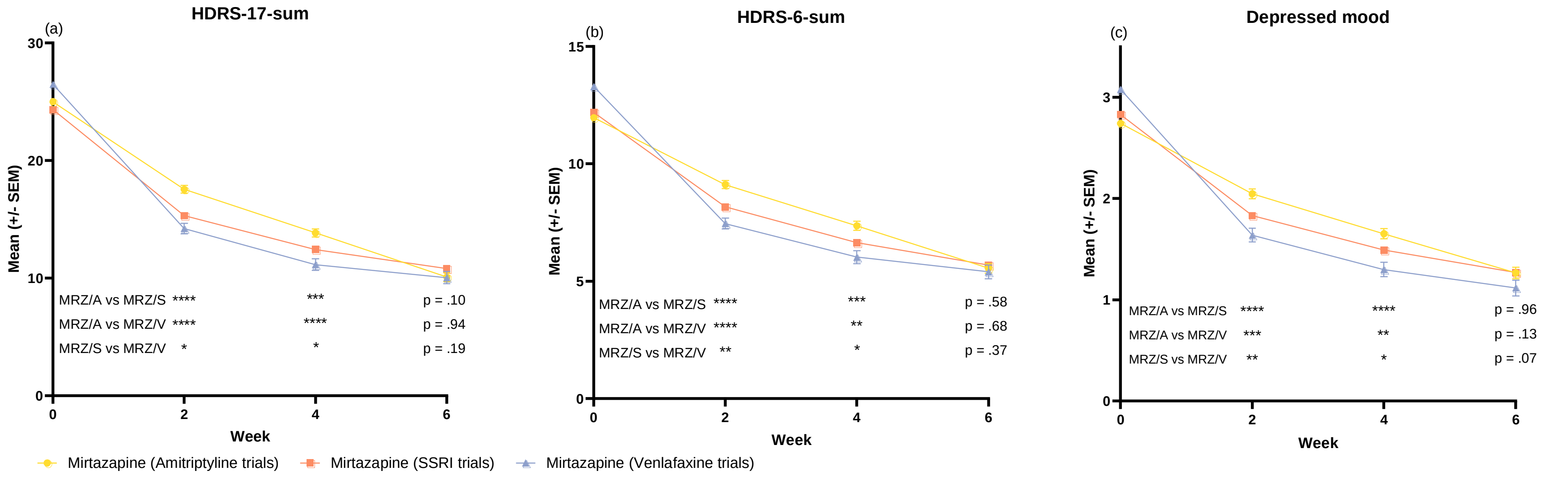

Shown are mean values for patients treated with mirtazapine in trials with amitriptyline, SSRIs, or venlafaxine as comparator, with p-values indicating mirtazapine vs mirtazapine comparisons. MRZ/A = outcome with mirtazapine in studies vs amitriptyline, MRZ/S = outcome with mirtazapine in studies vs SSRIs, MRZ/V = outcome with mirtazapine in studies vs venlafaxine. * < ·05, ** < ·01, *** < ·001, **** < ·0001

Supplementary figure 5. Item-wise separation for TCAs vs SSRIs and TCAs vs venlafaxine after two and six weeks of treatment in patients with a score of at least two on psychomotor retardation

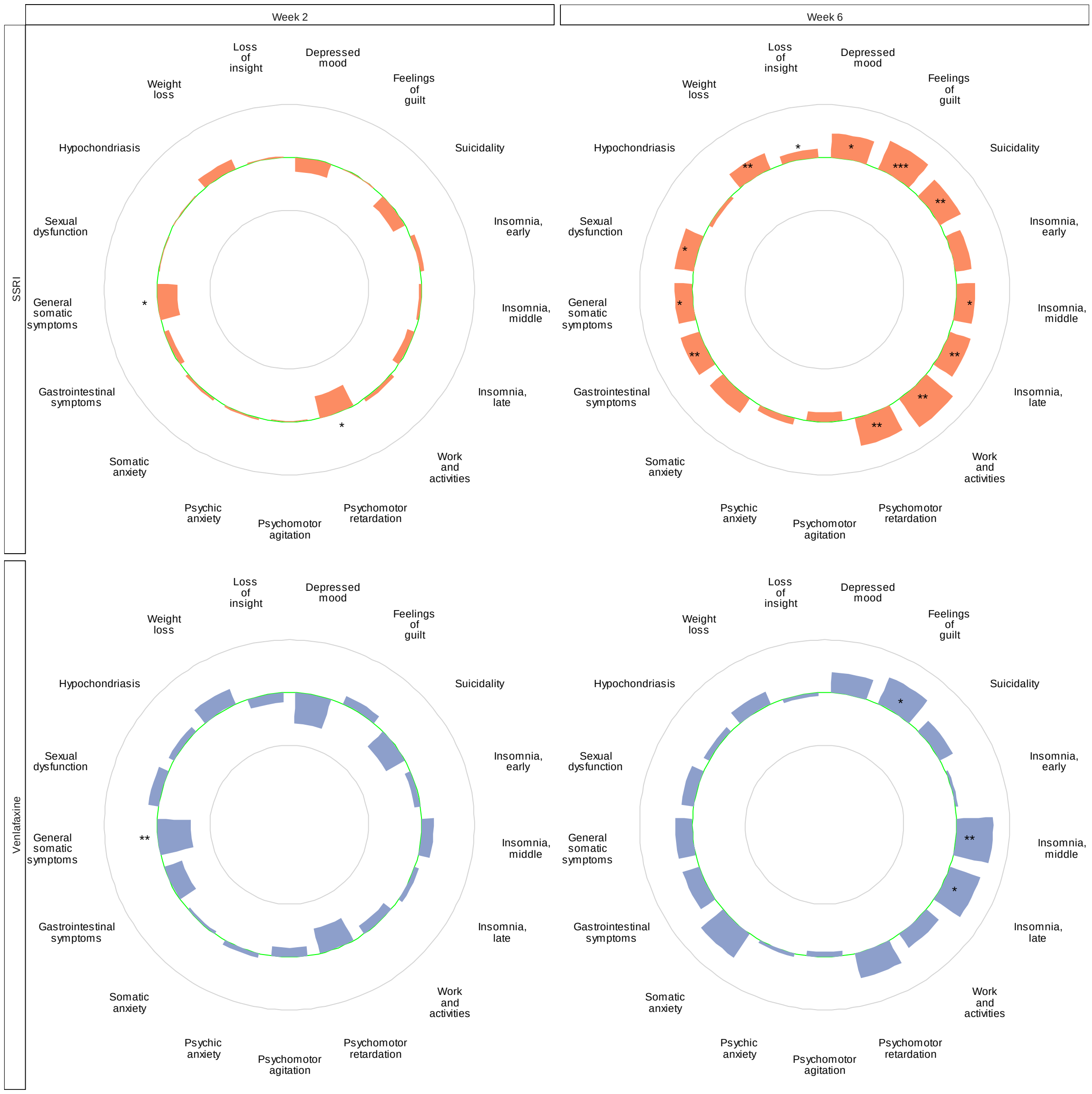

The green centre line represents no difference between mirtazapine and control. Bars pointing towards the outer circle favour the tricyclics, where a bar reaching the outer circle would correspond to a mean difference (MD) of +0·4 points. Bars pointing towards the inner circle favour the SSRI and venlafaxine, respectively, where a bar reaching the inner circle would correspond to a mean difference of -0·4 points. * = p < ·05, ** = p < ·01, *** = p < ·001, **** = p < ·0001.

Supplementary figure 6. Item-wise analyses of endpoint (week 6) differences using last observation carried forward-methodology

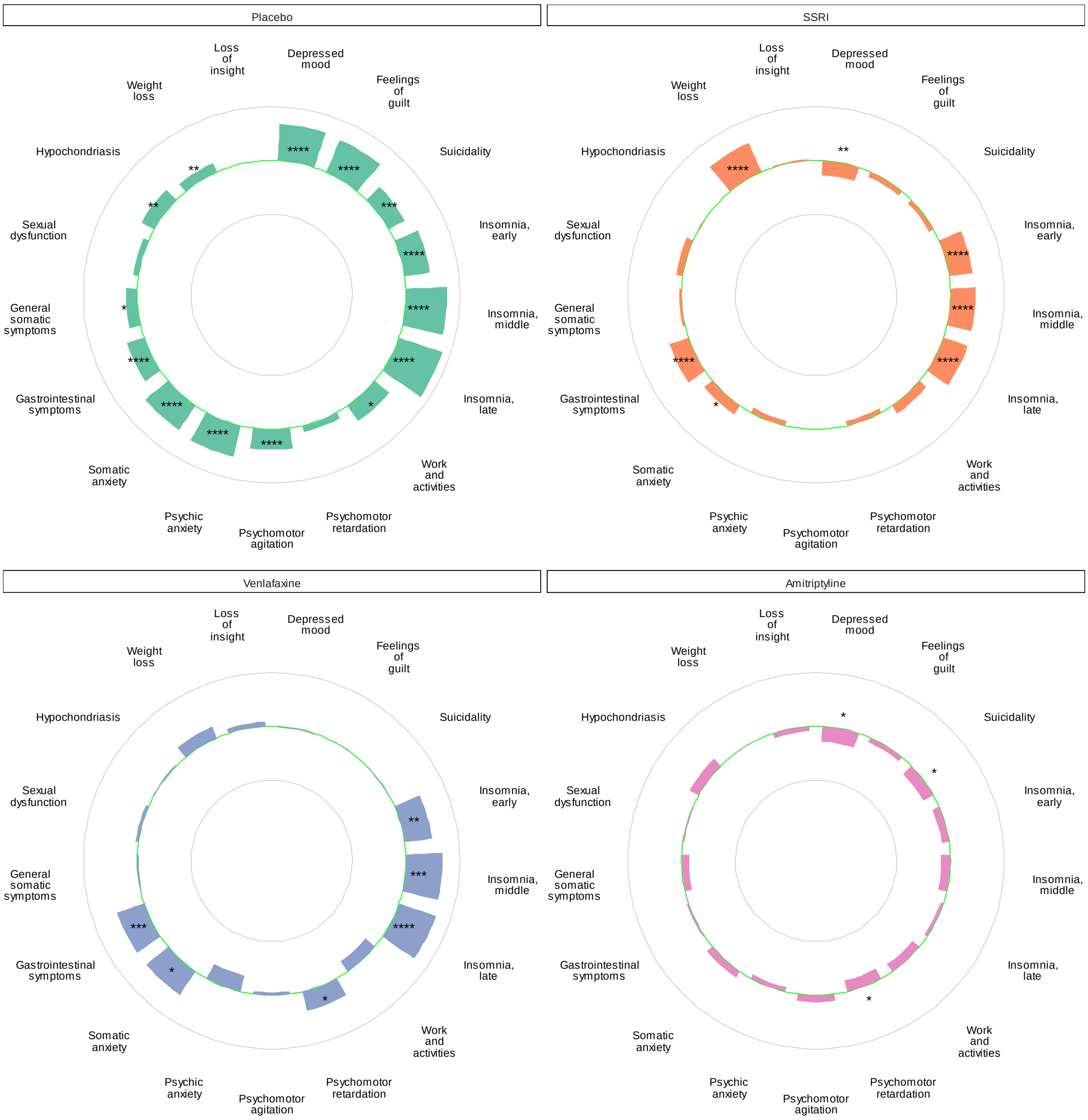

The green centre line represents no difference between mirtazapine and placebo or the active comparator. Bars pointing towards the outer circle favour the tricyclics, where a bar reaching the outer circle would correspond to a mean difference (MD) of +0·4 points. Bars pointing towards the inner circle favour the placebo or active comparator, respectively, where a bar reaching the inner circle would correspond to a mean difference of -0·4 points. * = p < ·05, ** = p < ·01, *** = p < ·001, **** = p < ·0001.
